# Extracts of Common Pesticidal Plants Increase Plant Growth and Yield in Common Bean Plants

**DOI:** 10.3390/plants9020149

**Published:** 2020-01-23

**Authors:** Angela G. Mkindi, Yolice L. B. Tembo, Ernest R. Mbega, Amy K. Smith, Iain W. Farrell, Patrick A. Ndakidemi, Philip C. Stevenson, Steven R. Belmain

**Affiliations:** 1 Department of Sustainable Agriculture, Biodiversity and Ecosystems Management, Centre for Research, Agricultural Advancement, Teaching Excellence and Sustainability (CREATES), The Nelson Mandela African Institution of Science and Technology, Box 447 Arusha, Tanzania; angela.mkindi@nm-aist.ac.tz (A.G.M.); ernest.mbega@nm-aist.ac.tz (E.R.M.); patrick.ndakidemi@nm-aist.ac.tz (P.A.N.); 2 Department of Crop and Soil Sciences, Lilongwe University of Agriculture and Natural Resources, Bunda, Malawi; ytembo@bunda.luanar.mw; 3 Royal Botanic Gardens, Kew, Richmond, Surrey TW9 3DS, UK; AmyKendall.Smith@kew.org (A.K.S.); I.Farrell@kew.org (I.W.F.); P.C.Stevenson@greenwich.ac.uk (P.C.S.); 4 Faculty of Biological Sciences, University of Leeds, Leeds LS2 9JT, UK; 5 Natural Resources Institute, University of Greenwich, Central Avenue, Chatham Maritime, Kent ME4 4TB, UK

**Keywords:** induced systemic response, foliar fertiliser, rutin, tryptophan, phenylalanine, botanicals

## Abstract

Common bean (*Phaseolus vulgaris*) is an important food and cash crop in many countries. Bean crop yields in sub-Saharan Africa are on average 50% lower than the global average, which is largely due to severe problems with pests and diseases as well as poor soil fertility exacerbated by low-input smallholder production systems. Recent on-farm research in eastern Africa has shown that commonly available plants with pesticidal properties can successfully manage arthropod pests. However, reducing common bean yield gaps still requires further sustainable solutions to other crop provisioning services such as soil fertility and plant nutrition. Smallholder farmers using pesticidal plants have claimed that the application of pesticidal plant extracts boosts plant growth, potentially through working as a foliar fertiliser. Thus, the aims of the research presented here were to determine whether plant growth and yield could be enhanced and which metabolic processes were induced through the application of plant extracts commonly used for pest control in eastern Africa. Extracts from *Tephrosia vogelii* and *Tithonia diversifolia* were prepared at a concentration of 10% *w/v* and applied to potted bean plants in a pest-free screen house as foliar sprays as well as directly to the soil around bean plants to evaluate their contribution to growth, yield and potential changes in primary or secondary metabolites. Outcomes of this study showed that the plant extracts significantly increased chlorophyll content, the number of pods per plant and overall seed yield. Other increases in metabolites were observed, including of rutin, phenylalanine and tryptophan. The plant extracts had a similar effect to a commercially available foliar fertiliser whilst the application as a foliar spray was better than applying the extract to the soil. These results suggest that pesticidal plant extracts can help overcome multiple limitations in crop provisioning services, enhancing plant nutrition in addition to their established uses for crop pest management.

## 1. Introduction

Common bean (*Phaseolus vulgaris*) is a strategic crop in low- and middle-income countries, known for its economic and nutritional benefits [1,2]. Tanzania is among the top 20 producers of common bean in the world [3]. However, bean productivity is generally stagnant across much of Africa due to a number of suboptimal provisioning services such as poor soil fertility and pest damage that are limiting potential yields [4,5]. Although chemical fertilizers can dramatically increase bean yields, they are largely unaffordable and unavailable to most smallholder farmers [6] and contribute to reduced soil stability [7,8], pollution [9] and carbon footprint [10]. Natural soil fertility enhancement through the use of manure, composts, green mulches, cover crops and crop rotation are not widely used by smallholder farmers, arguably due to high labour costs and poor local knowledge [11,12,13,14].

Sustainable technologies for pest management in legume crops often relies on the breeding of resistant varieties [15]. However, the use of pesticidal plant extracts in smallholder farming systems is also an established agro-ecologically sustainable pest control method [16,17,18,19,20]. Although the economics and cost-benefits of smallholder use of crude plant extracts for pest management are certainly favourable in many situations [19], uptake and promotion of pesticidal plants could be further facilitated by increased evidence on potential multiple benefits of their use [21], making their use even more attractive to smallholder farmers. For example, recent research has shown that the impact of pesticidal plants on beneficial arthropods such as pollinators and predators, is much less than that observed when using synthetic pesticides [18]. Research has also demonstrated that other potential benefits to smallholder use of pesticidal plants could be through direct effects on plant vigour by functioning as a green fertiliser or through the provision of additional nutrition and inducing systemic plant responses [22,23]. Very often plants used as pesticides have multiple uses such as providing fruits, seeds, fibre, timber or in traditional medicines [24,25,26]. Alternative uses can also include use as green mulches and cover crops to improve the soil fertility, where previous research points particularly to the use of *Tephrosia vogelii* and *Tithonia diversifolia* [27,28,29,30]. This study, therefore, sought to evaluate the contribution of extracts from *T. vogelii* and *T. diversifolia* on the growth, yield and metabolism of common beans. Evidence from this study could validate farmer observations and increase the perceived value of using such extracts, thus encouraging wider uptake in smallholder farming systems.

## 2. Results and Discussions

### 2.1. Growth and Yield of Common Beans in Response to the Application of Treatments

Extracts were applied to the leaves through foliar spraying or directly to soil as a soil drench in order to compare the effects on bean plant growth and yield. Significant variation in the growth of common beans was observed according to treatments where *T. vogelii* extracts resulted in significantly higher plant height, number of leaves and branches, leaf area, stem width and leaf greenness. However, water, water and soap and synthetic pesticide treatments were significantly lower in terms of plant height number of leaves, number of branches per plant, leaf area, stem width and leaf greenness (Table S1).

Yield was measured using the number of pods per plant and seed yield per plant (Table 1). Significantly higher numbers of pods and seeds were recorded in the *T. vogelii* treatment, followed by *T. diversifolia* and the foliar fertiliser for pods per plant and seed yield per plant. The control treatments (water, water and soap and synthetic pesticide) recorded significantly lower numbers for pods per plant and seed yield. Both number of pods per plant and seeds per pod showed a significant variation with respect to the method of application with higher values recorded for the number of seeds per pod and seed yield per plant when treated by foliar spray compared to when the treatments were applied to the soil for pod number and seed yield.

As the effect was much more pronounced when applied to the leaves compared to the soil, our data suggest that the plant extracts contribute to plant nutrition as a foliar fertiliser, which may be particularly useful in smallholder farming systems where soils are often degraded. Furthermore, these data suggest that previous reports on the use of these pesticidal plants in crop protection [17,18,31] have maintained crop yield not only by fighting pests, but by functioning as a foliar fertiliser. Contribution to growth and yield is likely to be related to the addition of nitrogen [32] where *T. diversifolia* [33,34] and *T. vogelii* [35,36,37] are known to produce nitrogen-rich green biomass.

### 2.2. Effect of Treatments and Application Method on Common Bean Metabolite Production

Analysis of chlorophyll content, flavonoids and anthocyanins indicated that the *T. vogelii* treatment resulted in significantly higher chlorophyll concentration, followed by the foliar fertiliser and *T. diversifolia* (Table 2). Lower chlorophyll content was observed in water, water and soap and the synthetic pesticide. Flavonoid content was highest in *T. diversifolia* treated plants, followed by the foliar fertiliser and *T. vogelii*, and these were significantly different from the water and water and soap treatments. Pereira et al. [38] reported that chlorophyll content could enhance photosynthesis rates, which ultimately influences plant vigour. No significant variation was observed in anthocyanin content across treatments or modes of application suggesting that the influence of treatments on plant metabolism was specific.

As expected, the commercial foliar fertiliser had a significant effect on metabolite production. The effect of *T. diversifolia* on chlorophyll content was supported by previous research by Oke et al. [39]. Leaf samples were further analysed to identify the contribution of treatments on the amounts of specific metabolites including primary metabolites (phenylalanine and tryptophan) and the secondary metabolite, rutin. An analysis of variance showed that these metabolites were higher when exposed to the foliar spray method of application in comparison with soil drenching (Table 3).

**Figure 1:**
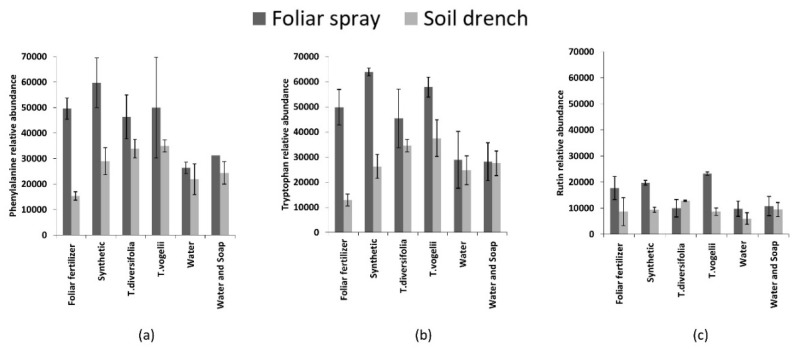
Relative abundance (mg/g dry weight) of (**a**) phenylalanine, (**b**) tryptophan and (**c**) rutin in common bean plants when exposed to different experimental treatments.

Overall, the foliar application was more effective in inducing changes, regardless of treatment (Figure 1). Foliar application was effective because it facilitated direct contact between the solution applied and the leaf surface where adsorption takes place [40,41], whereas application to the soil is indirect [36]. From this study, the production of amino acids induced by *T. diversifolia* and *T. vogelii* was similar to that observed with Neem (*Azadirachta indica*) where similar metabolic changes were reported by Sharma [42]. Similarly, Neem extracts applied to tomatoes have been observed to increase the abundance of several flavonoids through the jasmonate pathway [22].

Primary and secondary metabolites in plants can contribute to the development and growth of crop plants [22] as well as contribute to plant defence mechanisms [43]. Flavonoids are known to help a plant relate with other organisms and the environment thereby responding to biotic and abiotic stress [44,45]. Their contribution to growth is explained by their effect on auxin transport, shoot growth, root development and nitrogen fixing processes in legumes [46,47,48,49]. Examples of flavonoids in bean plants are kaempferol, quercetin [50,51], and rutin [52]. Flavonoids are also reported to mediate plant resistance to herbivores [53] thus, their increased occurrence could enhance defence against antagonists. Amino acids such as phenylalanine and tryptophan are known to contribute to plant growth and metabolism such as auxin biosynthesis in the rhizosphere [54], growth and nodulation [55]. Hence, applications that increase such metabolites in common beans could be beneficial to provide sustainable production techniques for bean resistance to pests, growth and yield as reported for ginger (*Zingiber officinale*) [56].

### 2.3. Correlations Between Bean Plant Growth Yield Parameters and Common Bean Metabolites

**Figure 2:**
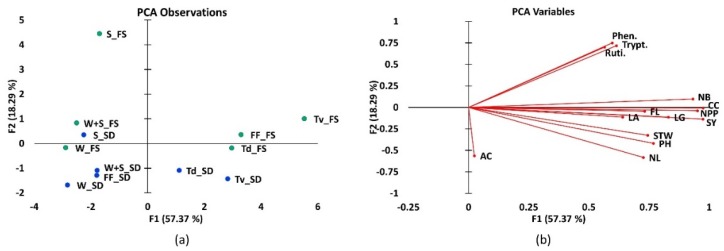
Two-dimensional principal component analysis (PCA) of (**a**) treatments applied using foliar spray and soil drench methods. Green marks indicate the treatments applied using foliar spray (FS) while blue marks indicate the application by soil drench (SD) where Tv = *T. vogelii*; Td = *T. diversifolia*; FF = foliar spray; W = water only; W + S = water and soap; S = synthetic; and (**b**) the covariance among all growth and metabolite parameters where CC = Chlorophyll content; FL = Flavonoids; AN = Anthocyanins; PH = Plant height; NL = Number of leaves; NB = Number of branches; LA = Leaf area; SW = Stem width; LG = Leaf greenness; NPP = Number of pods per plant; and SY = seed yield/plant.

Three principal components (PC1, PC2 and PC3) were retained to explain 87.2% variance of the dependent variables (Table S1). The criteria for selection were based on a cumulative variance of 70% and an eigenvalue greater than one. The first principal component accounted for a total variance of 57.37%, while the second and third components explained 18.3% and 8.7% of the total variance, respectively. PCA observations of the treatments and their modes of application indicated the plant extracts applied to the bean plant or the soil were grouped together, implying that their contribution to bean growth was related (Figure 2a). Regardless of the plant extract species, application to the leaves had a negative relation with application to the soil. *T. vogelii* (Foliar spray) and water (Soil drench) were the treatments showing the highest and lowest influence, respectively. Furthermore, applying water had a low effect on the bean crop development regardless of the method of application.

Anthocyanin content correlated with the second principal component, which was different from the rest of the variables that all correlated with the first principal component (Figure 2b). This difference is likely to be based on the fact that anthocyanin values were minimal across all the treatments, with no significant difference observed in influencing bean development across the treatments. The first principal component’s interpretation showed that yield parameters (number of pods per plant and seed yield per plant) and chlorophyll content explained more of the variation describing effects of the treatments. The number of branches showed a positive correlation with key metabolites, e.g., rutin (0.61), phenylalanine (0.58) and tryptophan (0.63). The PCA correlation matrix, eigenvalues, factor loadings, and factor scores at *p* = 0.05 can be found in Tables S2–S6.

## 3. Materials and Methods

### 3.1. Bean Rearing and Plant Material Preparation

The experiment was carried out in a controlled pest-free glass house at the Nelson Mandela African Institution of Science and Technology, Arusha, Tanzania (Latitude 3°24′ S Longitude 36°47′ E, elevation of 1168 masl with a mean annual rainfall of 1200 mm, mean maximum temperature of 21.7 °C and mean minimum temperature of 13.6 °C). Each treatment unit consisted of eight bean plants. Common bean seeds used for the experiment were of Lyamungo 90 variety, purchased from the Seliani Agricultural Research Institute. Two seeds were planted in each pot, later thinned to one plant per each pot, using 2-litre volume pots containing standard potting compost. All pots were arranged in a complete randomized block design on a bench in the glasshouse, providing even lighting, ventilation, temperature (25 ± 5 °C) and equal amounts of water per pot.

Pesticidal plant materials (*T. vogelii* and *T. diversifolia*) were collected from Lyamungo field areas, dried under the shade and ground into fine powder using previously reported methods and locations [18]. *T. vogelii* and *T. diversifolia* are among a large group of insecticidal plants that have been used for decades for pest control [17,18,19,57]. Positive controls included synthetic pesticide (Karate, lambda cyhalothrin) and a commercial foliar fertiliser (BioForce, an organic extract from seaweeds and blue green algae) which were applied according to instructions provided on the label. Pesticidal plant powders were extracted in soapy water (0.1% soap) to produce an extract solution of 10% (*w/v*) following previously reported methods [18]. Negative control treatments were with plain water, and water with 0.1% soap.

All treatments were applied in two different methods, either as a foliar spray using a hand sprayer or directly to the soil with a small watering can, ensuring equal amounts were applied to each plant. The treatments were applied fortnightly from the first week after plant germination until the time of bean flowering, i.e., a total of four treatment applications.

### 3.2. Collection of Growth Parameters Data and Leaf Samples for Chemical Analysis

Growth parameters and samples for chlorophyll content and bean leaf chemistry analysis were collected before bean flowering. Yield parameters were collected close to the maturity of the beans and the total yield collected after final bean harvesting. The growth parameters that were measured included plant height, number of leaves, number of branches, main stem width, leaf area and leaf greenness. Leaf greenness was scored using a scale of 1–5 where 1 was regarded as low greenness and 5 as high greenness using a leaf colour chart as previously reported [58]. Leaf area was determined from the direct measurements of length as a distance between the base and apex of the leaflet, and the width between positions of the leaflets. Leaf area was then calculated using the formula described by Bhatt [59]

LA = 11.98 + 0.06 L × W(1)

where LA = Leaf area’, L = leaf length and W = leaf width.

Plant leaf samples were harvested three days after spraying the beans. Harvesting was done at the vegetative stage, just before flowering. Four plants from each treatment were randomly selected from each plant. The leaves were thoroughly washed with distilled water. Two leaves from each plant were placed in a desiccator with silica gel, desiccated and prepared for phytochemical analysis. The other two leaves collected from each plant were used for spectrophotometric analysis described below.

### 3.3. Spectrophotometric Analysis of Key Metabolite Groups in Bean Leaves

#### 3.3.1. Chlorophyll Content Analysis

Chlorophyll concentration was determined through the extraction of chlorophyll from the third leaf of the growing tip of each plant using Dimethyl Sulphoxide (DMSO) as described by Hiscox, 1980 [60]. This involved placing 100 mg of the middle portion of the leaf in a 15 mL vial containing 7 mL DMSO and incubating at 65 °C for 24 h after which the leaves were completely transparent signifying chlorophyll extraction. The extracted liquid was transferred to graduated tubes and made up to a total volume of 10 mL with DMSO and then kept at 4 °C waiting for analysis. To determine the chlorophyll content, 300 microliters of the sample were transferred into an 86-well plate, where the absorbance at 645 nm and 663 nm were read using a spectrophotometer (Synergy, Multi-mode reader, Biotek Instrument Inc. Winooski, VT, USA) against DMSO as a blank. Chlorophyll levels in milligrams per litre (mg/l) were then calculated using the formula described by Arnon [61].

Total Chl = 20.2 × D645 nm + 8.02 × D663 nm(2)

where Chl = Chlorophyll, D = the Absorbance value at the respective wavelengths obtained from the spectrophotometer.

#### 3.3.2. Anthocyanins and Flavonoids Analysis

Flavonoids and anthocyanins in bean plant leaves were determined using the method described by Makoi et al. [62]. Dried and ground bean leaves were used, where 0.1 g of the plant powder was extracted in 10 mL acidified methanol, made at a ratio of 79:20:1 of MeOH:H_2_O:HCl. The extract was incubated for 72 h in darkness for auto extraction and then filtered through a filter paper (Whatman #2). Absorbance of the clear supernatant was measured at 300, 530, and 657 nm in a spectrophotometer (Synergy, Multi-mode reader, Biotek Instrument Inc. Winooski, VT, USA) against acidified methanol as a standard. Flavonoid concentration was obtained from the measured absorption at 300 nm and expressed in Abs g DM^−1^.

Abs g^−1^ DM = Abs300 (3)

Anthocyanins were measured by using the formula described by Lindoo and Caldwell [63].

Abs g^−1^ DM = Abs530 − 1/3 × Abs657 (4)

where Abs = Absorption readings recorded from the spectrophotometer. The resulting concentration was expressed as Abs g DM^−1^.

### 3.4. HPLC Detection of Primary and Secondary Metabolites

Desiccated beans leaves were powdered using an electric coffee grinder, and 50 mg of the powder was extracted in methanol (1 mL) and left to stand for 24 h at room temperature before chemical analysis. Extracts were transferred to Eppendorf tubes and centrifuged for 20 min at 500 rpm. From this 300 µL supernatant was transferred into HPLC glass vials for separation. The sample analyses were performed by Liquid Chromatography-Electrospray Ionization Mass Spectroscopy (LC-ESIMS) and UV spectroscopy using a Thermo Fisher Velos Pro LC-MS. Aliquots of extract were injected directly onto a Phenomenex (Macclesfield, Cheshire, UK) Luna C18(2) columns (150 × 3.0 mm i.d., 5 um particle size) and the compounds were eluted using methanol (A), water (B) and acetonitrile containing 1% formic acid (C) with A = 0%, B = 90% at T = 0 min; A = 90%, B = 0% at T = 20 min and held for 10 min with C at 10% throughout the analyses. Column temperature was 30 °C with flow rate = 0.5 mL min^−1^. High resolution MS spectra were used to provide additional data for compound identification and were recorded for a subset of samples using a Thermo LTQ-Orbitrap XL mass spectrometer (Waltham, MA, United States) with compound separation on an Accela LC system.

### 3.5. Statistical Analysis

The experiment was conducted following the completely randomised block design with eight replications to assess yield and growth of common beans and four replications to assess the metabolites. Effects of treatments and their interactions observed were subjected to Analysis of Variance. The means of treatments and interactions were compared using the least significant difference (LSD) test at a significant level of *p* ≤ 0.05. Principal Component Analysis (PCA) was performed to explain potential covariance between bean plant growth, yield parameters and common bean metabolites. All the analyses were done using XLSTAT version 2019.2.2.59614 (Addinsoft (2019). XLSTAT statistical and data analysis solution. Boston, MA, USA. https://www.xlstat.com).

## 4. Conclusions

In this study, foliar sprays of the pesticidal plants *T. vogelii* and *T. diversifolia* enhanced common bean growth, yield and induced essential metabolites known for facilitating plant growth. Thus, their use helps to reduce the need for both synthetic pesticides and fertilisers by sustainably reducing arthropod pests whilst increasing plant nutrition. As soil fertility and crop pests are considered two of the main problems contributing to the yield gaps of smallholder farmers, using botanical extracts for crop production can help farmers move towards more sustainable agro-ecological approaches to crop production, tackling two problems at the same time. Pesticidal plants such as *T. vogelii* and *T. diversifolia* can be obtained cheaply in many African countries. *T. vogelii* can easily be propagated, although it should not be cultivated near large bodies of water as the rotenoid compounds can be harmful to fish. *T. diversifolia* is widely growing in roadsides and field margins and is considered invasive in some parts of Africa, therefore, care is also needed when cultivating this plant to keep it under control. Other commonly used pesticidal plant species may also have beneficial impacts on crop growth, where further validation is recommended.

## Figures and Tables

**Table 1:** Effects of foliar fertiliser, synthetic and plant pesticide treatments and application method on the yield of common beans.

Treatment Applied	Number of Pods Per Plants	Seed Yield/Plant (g)
Foliar Fertiliser	3.1 ± 0.26 b	2.7 ± 0.33 b
Synthetic pesticide	2.1 ± 0.24 c	1.3 ± 0.19 c
*Tephrosia vogelii*	4.1 ± 0.23 a	3.8 ± 0.23 a
*Tithonia diversifolia*	3.1 ± 0.31 b	3.3 ± 0.23 b
Water	1.9 ± 0.23 c	1.5 ± 0.16 c
Water and soap	1.6 ± 0.22 c	1.7 ± 0.11 c
**Method of Application**		
Foliar spray	2.9 ± 0.21 a	2.7± 0.20 a
Soil drenching	2.4 ± 0.16 b	2.1± 0.16 b
**2-Way ANOVA (F-Statistics)**		
Treatment	15.2 ***	29.0 ***
Treatment method	6.7 *	14.8 ***
Treatment * Treatment method	2.0 *	3.1 *

The values presented are means ± SE. *, *** = significant at *p* ≤ 0.05, *p* ≤ 0.001 respectively. Means followed by the same letter in a column are not significantly different.

**Table 2:** Effect of treatment on the presence of key metabolite groups in common bean.

Treatments	Chlorophylls (mg/L)	Flavonoids (Abs g DM^−1^)	Anthocyanins (Abs g DM^−1^)
Foliar fertiliser	19.3 ± 1.84 b	2.8 ± 0.28 ab	0.1 ± 0.01 a
Synthetic pesticide	13.7 ± 0.74 c	2.4 ± 0.14 bcd	0.1 ± 0.00 a
*Tephrosia vogelii*	24.6 ± 1.29 a	2.7 ± 0.23 abc	0.1 ± 0.01 a
*Tithonia diversifolia*	18.9 ± 0.89 b	3.0 ± 0.16 a	0.1 ± 0.01 a
Water	12.7 ± 0.53 c	2.1 ± 0.17 d	0.1 ± 0.03 a
Water and soap	14.0 ± 0.49 c	2.2 ± 0.15 cd	0.1 ± 0.02 a
**Method of Application**			
Soil drench	15.9 ± 0.89 b	2.5 ± 0.12 a	0.1 ± 0.01 a
Foliar spray	18.5 ± 1.14 a	2.6 ± 0.13 a	0.1 ± 0.01 a
**2-Way ANOVA (F-Statistics)**			
Treatment	27.8 ***	3.4 *	0.6ns
Method of application	12.7 **	0.5ns	0.4ns
Treatment * Method of application	3.0 *	1.3ns	0.3ns

The values presented are means ± SE. *, **, *** = significant at *p* ≤ 0.05, *p* ≤ 0.01, *p* ≤ 0.001 respectively, ns = not significant. Means followed by the same letter in a column are not significantly different.

**Table 3:** Two-way Analysis of Variance on the influence of mode of application on the relative abundance (mg/g dry weight) of phenylalanine, tryptophan and rutin.

Method of Application	Phenylalanine	Tryptophan	Rutin
Foliar spray	43608.3 ± 4557.06 a	45478.3 ± 5450.15 a	15093.8 ± 1675.05 a
Soil drench	26209.9 ± 2127.52 b	26805.8 ± 2566.88 b	9342.5 ± 895.06 b
Two-way ANOVA (F-statistics)	13.4 ***	10.3 **	12.8 ***

The values presented are means ± SE. **, *** = significant at *p* ≤ 0.01, *p* ≤ 0.001 respectively. Means followed by the same letter in a column are not significantly different.

## Supplementary Materials

The following are available online at https://www.mdpi.com/2223-7747/9/2/149/s1, Table S1: Effects of foliar fertiliser, synthetic pesticides and botanical plants extract on common beans growth. The values presented are means ± SE. *, **, *** = significant at *p* ≤ 0.05, *p* ≤ 0.01, *p* ≤ 0.001 respectively, ns = not significant. Means followed by the same letter in a column are not significantly different. Tables S2–S6: The correlation matrix used, eigenvalues, factor loadings, and factor scores.
