# Supplementary material for: Extracts of Common Pesticidal Plants Increase Plant Growth and Yield in Common Bean Plants
Source: Plants (Basel). 2020 Jan 23;9(2):149. doi: 10.3390/plants9020149 (PMC7076451; doi:10.3390/plants9020149)
Supplement: Supplementary file 1 [file plants-09-00149-s001.pdf]

Supplementary Table S1. Effects of foliar fertilizer, synthetic pesticide and botanical plant extracts on common bean growth. The values presented are means ± SE. *, **, *** = significant at P ≤ 0.05, P ≤ 0.01, P ≤ 0.001, respectively, ns=not significant. Means followed by the same letter in a column are not significantly different.

Treatments	Plant height (cm)	Number of leaves	Number of branches	Leaf area	Stem width (mm)	Leaf greenness
**Treatment applied**						
Foliar fertilizer	36.7 ± 1.17 a,b	3.1 ± 0.13 a,b	2.9 ± 0.21 b,c	21.4 ± 0.38 a,b	3.7 ± 0.08 a	3.2 ± 0.21 b
Synthetic Pesticide	33.2 ± 1.20 c	2.7 ± 0.17 c	2.6 ± 0.22 b,c	20.4 ± 0.49 b	3.3 ± 0.09 b	3.3 ± 0.27 b
Tephrosia vogelii	39.6 ± 1.38 a	3.2 ± 0.17 a	3.6 ± 0.18 a	22.6 ± 0.58 a	3.7 ± 0.10 a	4.8 ± 0.13 a
Tithonia diversifolia	36.4 ± 0.89 b	3.2 ± 0.14 a	3.0 ± 0.11 b	21.6 ± 0.72 a,b	3.7 ± 0.10 a	4.5 ± 0.16 a
Water	34.1 ± 0.88 b,c	3.0 ± 0.00 a,b,c	2.5 ± 0.13 c	21.5 ± 6.0.37 a,b	3.3 ± 0.07 b	3.0 ± 0.19 b
Water and Soap	36.2 ± 1.20 b,c	2.8 ± 0.09 b,c	2.6 ± 0.12 b,c	21.3 ± 0.71 a,b	3.4 ± 0.08 b	3.3 ± 0.25 b
**Method of application**						
Foliar spray	35.8 ± 0.81 a	3.0 ± 0.07 a	3.0 ± 0.12 a	21.9 ± 0.36 a	3.5 ± 0.06 a	3.7 ± 0.17 a
Soil drench	36.3 ± 0.58 a	3.0 ± 0.09 a	2.8 ± 0.09 a	21.0 ± 0.28 b	3.5 ± 0.05 a	3.7 ± 0.14 a
**2 way ANOVA (F statistics)**						
Treatment	4.26 **	2.38 *	5.56 ***	1.86 **	5.70 ***	12.36 ***
Method of application	0.36 ns	0.60 ns	2.26 ns	4.16 *	1.16 ns	0.30 ns
Treatment*Method of application	2.71 *	0.60 ns	0.31 ns	3.52 **	1.00 ns	0.89 ns

Supplementary Table S2. Effects of foliar fertilizer, synthetic pesticide and botanical plant extracts on common bean growth. Correlation matrix (Pearson (n)): CC = Chlorophyll content; FL = Flavonoids; AN = Anthocyanins; PH = Plant height; NL = Number of leaves; NB = Number of branches; LA = Leaf area; SW = Stem width; LG = Leaf greenness; NPP = Number of pods per plant; and SY=seed yield/plant; Phen= Phenylalanine; Trypt=Tryptophan and Ru=Rutin.

Variables	CC	FL	AC	PH	NL	NB	LA	STW	LG	NPP	SY	Phen.	Trypt.	Ru.
CC	**1**													
FL	**0.715**	**1**												
AC	0.013	−0.038	**1**											
PH	**0.772**	0.452	0.188	**1**										
NL	**0.690**	**0.682**	0.489	**0.707**	**1**									
NB	**0.936**	0.575	−0.014	**0.654**	**0.608**	**1**								
LA	**0.597**	0.133	0.186	**0.683**	0.374	0.560	**1**							
STW	**0.718**	**0.691**	−0.050	**0.668**	**0.738**	**0.620**	0.251	**1**						
LG	**0.754**	0.483	−0.039	**0.652**	**0.604**	**0.815**	**0.583**	**0.661**	**1**					
NPP	**0.942**	**0.678**	−0.012	**0.716**	**0.687**	**0.887**	**0.689**	**0.671**	**0.781**	**1**				
SY	**0.956**	**0.738**	0.070	**0.781**	**0.784**	**0.884**	**0.653**	**0.762**	**0.814**	**0.938**	**1**			
Phen.	0.559	0.461	−0.257	0.131	0.022	**0.583**	0.323	0.151	0.402	0.515	0.471	**1**		
Trypt.	0.574	0.379	−0.141	0.167	0.066	**0.631**	0.375	0.132	0.425	0.527	0.478	**0.980**	**1**	
Ru.	0.548	0.353	−0.302	0.158	0.088	**0.608**	0.201	0.250	0.272	0.471	0.451	**0.815**	**0.846**	**1**

Values in bold are different from 0 with a significance level alpha=0.05.

Supplementary table S3. Eigenvalues.

	F1	F2	F3	F4	F5	F6	F7	F8	F9	F10	F11
Eigenvalue	8.032	2.560	1.199	0.915	0.382	0.362	0.222	0.163	0.095	0.047	0.024
Variability (%)	57.374	18.286	8.566	6.535	2.727	2.587	1.585	1.161	0.677	0.332	0.171
Cumulative %	57.374	75.660	84.226	90.761	93.487	96.074	97.659	98.820	99.497	99.829	100.000

Supplementary material S4. Factor loadings and correlations between variables and factors CC = Chlorophyll content; FL = Flavonoids; AN = Anthocyanins; PH = Plant height; NL = Number of leaves; NB = Number of branches; LA = Leaf area; SW = Stem width; LG = Leaf greenness; NPP = Number of pods per plant; SY=seed yield/plant; Phen= Phenylalanine; Trypt=Tryptophan and Ru=Rutin.

	F1	F2	F3	F4	F5	F6	F7	F8	F9	F10	F11
CC	0.975	−0.007	−0.019	−0.005	−0.089	0.011	−0.102	0.114	0.116	−0.029	−0.056

FL	0.732	−0.045	−0.494	0.301	0.068	0.340	0.013	0.041	−0.047	−0.025	0.052
AC	0.024	−0.562	0.493	0.650	0.036	−0.096	0.049	−0.005	0.069	−0.006	−0.002
PH	0.768	−0.418	0.151	−0.201	−0.281	0.035	0.197	0.220	−0.056	0.040	−0.002
NL	0.726	−0.581	−0.100	0.307	0.006	−0.058	−0.042	−0.064	−0.141	0.041	0.000
NB	0.932	0.097	0.051	−0.037	0.053	−0.223	−0.188	0.128	0.050	−0.038	0.092
LA	0.640	−0.113	0.643	−0.291	−0.056	0.208	0.024	−0.169	−0.011	−0.038	0.052
STW	0.744	−0.325	−0.457	−0.088	−0.084	−0.151	0.204	−0.173	0.148	0.005	0.021
LG	0.831	−0.113	0.037	−0.266	0.411	−0.190	0.107	0.016	−0.089	0.007	−0.017
NPP	0.951	−0.039	0.040	−0.090	0.020	0.114	−0.202	−0.076	0.048	0.145	−0.025
SY	0.974	−0.137	−0.018	−0.019	0.001	0.054	−0.061	−0.054	−0.028	−0.127	−0.073
Phen.	0.597	0.749	0.084	0.165	0.102	0.124	0.141	0.021	0.037	0.001	−0.006
Trypt.	0.614	0.718	0.206	0.209	0.072	−0.012	0.118	0.019	0.024	0.040	−0.008
Ru.	0.565	0.701	−0.041	0.151	−0.301	−0.221	−0.031	−0.102	−0.117	−0.006	0.001

Supplementary material S5. Squared cosines of the variables: CC = Chlorophyll content; FL = Flavonoids; AN = Anthocyanins; PH = Plant height; NL = Number of leaves; NB = Number of branches; LA = Leaf area; SW = Stem width; LG = Leaf greenness; NPP = Number of pods per plant; and SY=seed yield/plant; Phen= Phenylalanine; Trypt=Tryptophan and Ru=Rutin.

	F1	F2	F3	F4	F5	F6	F7	F8	F9	F10	F11
CC	**0.951**	0.000	0.000	0.000	0.008	0.000	0.010	0.013	0.014	0.001	0.003
FL	**0.535**	0.002	0.244	0.091	0.005	0.116	0.000	0.002	0.002	0.001	0.003
AC	0.001	0.316	0.243	**0.423**	0.001	0.009	0.002	0.000	0.005	0.000	0.000
PH	**0.589**	0.175	0.023	0.040	0.079	0.001	0.039	0.048	0.003	0.002	0.000
NL	**0.527**	0.338	0.010	0.094	0.000	0.003	0.002	0.004	0.020	0.002	0.000
NB	**0.869**	0.010	0.003	0.001	0.003	0.050	0.035	0.016	0.003	0.001	0.009
LA	0.410	0.013	**0.414**	0.084	0.003	0.043	0.001	0.029	0.000	0.001	0.003
STW	**0.554**	0.106	0.209	0.008	0.007	0.023	0.042	0.030	0.022	0.000	0.000
LG	**0.690**	0.013	0.001	0.071	0.169	0.036	0.012	0.000	0.008	0.000	0.000
NPP	**0.905**	0.002	0.002	0.008	0.000	0.013	0.041	0.006	0.002	0.021	0.001
SY	**0.949**	0.019	0.000	0.000	0.000	0.003	0.004	0.003	0.001	0.016	0.005
Phen.	0.357	**0.561**	0.007	0.027	0.010	0.015	0.020	0.000	0.001	0.000	0.000

Trypt.	0.377	**0.515**	0.042	0.044	0.005	0.000	0.014	0.000	0.001	0.002	0.000
Ru.	0.319	**0.492**	0.002	0.023	0.091	0.049	0.001	0.010	0.014	0.000	0.000

Values in bold correspond for each variable to the factor for which the squared cosine is the largest.

Supplementary material S6. Squared cosines of the observations: FS= Foliar Spray, SD=Soil Drench.

	**F1**	**F2**	**F3**	**F4**	**F5**	**F6**	**F7**	**F8**	**F9**	**F10**	**F11**
Foliar fertilizer_FS	**0.697**	0.008	0.009	0.105	0.088	0.092	0.000	0.000	0.001	0.000	0.000
Foliar fertilizer_SD	**0.352**	0.184	0.239	0.101	0.019	0.009	0.037	0.035	0.021	0.000	0.002
Synthetic _FS	0.123	**0.843**	0.001	0.017	0.006	0.004	0.000	0.000	0.004	0.001	0.000
Synthetic _SD	**0.598**	0.014	0.135	0.162	0.000	0.033	0.001	0.008	0.026	0.023	0.001
T.diversifolia _FS	**0.692**	0.003	0.032	0.077	0.099	0.061	0.012	0.023	0.000	0.001	0.000
T.diversifolia _SD	0.118	0.114	**0.409**	0.263	0.034	0.038	0.014	0.001	0.007	0.001	0.000
T.vogelii _FS	**0.891**	0.029	0.014	0.021	0.010	0.031	0.001	0.001	0.002	0.000	0.000
T.vogelii _SD	**0.674**	0.174	0.009	0.000	0.039	0.001	0.021	0.070	0.011	0.001	0.000
Water _FS	**0.669**	0.003	0.134	0.080	0.007	0.004	0.085	0.006	0.012	0.001	0.000
Water _SD	**0.596**	0.212	0.115	0.020	0.002	0.003	0.031	0.003	0.009	0.002	0.005
Water and soap_FS	**0.702**	0.076	0.057	0.099	0.007	0.001	0.002	0.022	0.000	0.031	0.003
Water and soap_SD	**0.443**	0.166	0.266	0.004	0.036	0.015	0.032	0.009	0.008	0.001	0.018

Values in bold correspond for each observation to the factor for which the squared cosine is the largest.
